# Neglecting the Already Neglected – Do All Lives and All Morbidities Matter in Global Health?

**DOI:** 10.1002/hsr2.73476

**Published:** 2026-11-10

**Authors:** Robert Adamu Shey, Gordon Takop Nchanji, Yanick Aqua Stong Tangan, Melvis Berinyuy Dulandzev, Methodius Shinyuy Lahngong, Francis Nongley Nkemngo, Cabirou Mounchili Shintouo, Ntang Emmaculate Yaah, Bernis Neneyoh Yengo, Stephen Mbigha Ghogomu

**Affiliations:** ^1^ Department of Biochemistry and Molecular Biology University of Buea Buea Cameroon; ^2^ Tropical Disease Interventions, Diagnostics, Vaccines and Therapeutics (TroDDIVaT) Initiative Frontline Association Tiko Cameroon; ^3^ Department of Microbiology and Parasitology University of Buea Buea Cameroon; ^4^ Center for Infection Biology and Tropical Health Buea Cameroon

## Abstract

**Background and Aims:**

Neglected tropical diseases (NTDs) disproportionately affect the poorest communities, especially in Africa, yet global health strategies have consistently overlooked the significant mental health morbidity associated with these conditions. Despite the visible physical impairments, the associated mental and psychosocial burden, with depression and anxiety pooled prevalence estimated at around 38% in lymphoedema‐related NTDs (leprosy, podoconiosis, lymphatic filariasis), and individual studies reporting anywhere from roughly 10% to over 70% depending on disease and setting, remains inadequately addressed in policy and practice.

**Argument:**

This Viewpoint highlights the persistent mismatch between current global health rhetoric (particularly the goals of equity and inclusiveness) and on‐the‐field practices in NTD control efforts. Unlike previous commentaries and perspectives that have treated NTD‐associated mental health as an add‐on service gap, we situate this neglect within a broader critique of the current structural priorities in global health, viewed through an African lens, and argue that this constitutes a major blind spot that can undermine the progress toward the WHO NTD Roadmap (2021–2030) and Sustainable Development Goal 3 (SDG3).

**Evidence:**

NTD sufferers experience anxiety, depression, post‐traumatic stress disorder (PTSD), social exclusion, chronic pain, and reduced quality of life in addition to physical debilitation. Despite the huge mental health burden, an estimated 76%–85% of people with mental disorders in LMICs receive no treatment. None of the five key NTD intervention pillars formally incorporates a mental health component, though some national programs include informal psychosocial elements.

**Recommendations:**

Building on the current evidence gap, and for funding agencies, policymakers, and program managers, we propose concrete integration steps, notably routine mental health screening using validated brief tools (e.g., PHQ‐9, WHODAS 2.0, and mhGAP) within community‐based NTD control programs and MDA campaigns, alongside training community health workers (CHWs) to provide basic psychosocial support; the inclusion of mental health indicators within national NTD monitoring frameworks; the development and implementation of policies that prioritize stigma reduction and person‐centered care; and increased funding for cross‐cutting research.

**Conclusion:**

Eliminating NTDs as a public health problem will not, by itself, resolve their heavy mental health consequences. Integrating mental health into NTD programming is a vital prerequisite for universal health coverage, reduced health inequity, achievement of the WHO NTD Roadmap (2021–2030), SDG3, and the “*health for all*” vision.

## 1 Introduction

Global health initiatives have, over the past few decades, achieved tremendous progress, from the near‐eradication of polio to the drastic reduction in HIV/AIDS mortality [1]. Recently, the global health community also witnessed the rallying of efforts against the COVID‐19 pandemic. However, these successes have been unevenly distributed, with certain diseases and certain groups of disease burden remaining persistently overlooked or ignored. Neglected tropical diseases (NTDs), a diverse group of 21 conditions affecting approximately 1 billion people, with 1.4 billion people requiring intervention [2], exemplify this paradox. Despite their enormous public health and socio‐economic burden, especially on the world's poorest communities, they have continued to receive just a tiny fraction of the attention and resources allocated to diseases affecting wealthier populations. Africa bears a disproportionate share of this burden, accounting for approximately 40% of the global NTD burden, and over 600 million individuals (predominantly in its poorest and most marginalized communities) require treatment [3]. Even more concerning is a new neglect within the neglect: this refers to the profound mental health morbidity experienced by those living with NTDs, which remains mostly invisible in global health discourses and programming (Figure 1), particularly in African and other LMIC contexts where mental health infrastructure is most limited.

**Figure 1:**
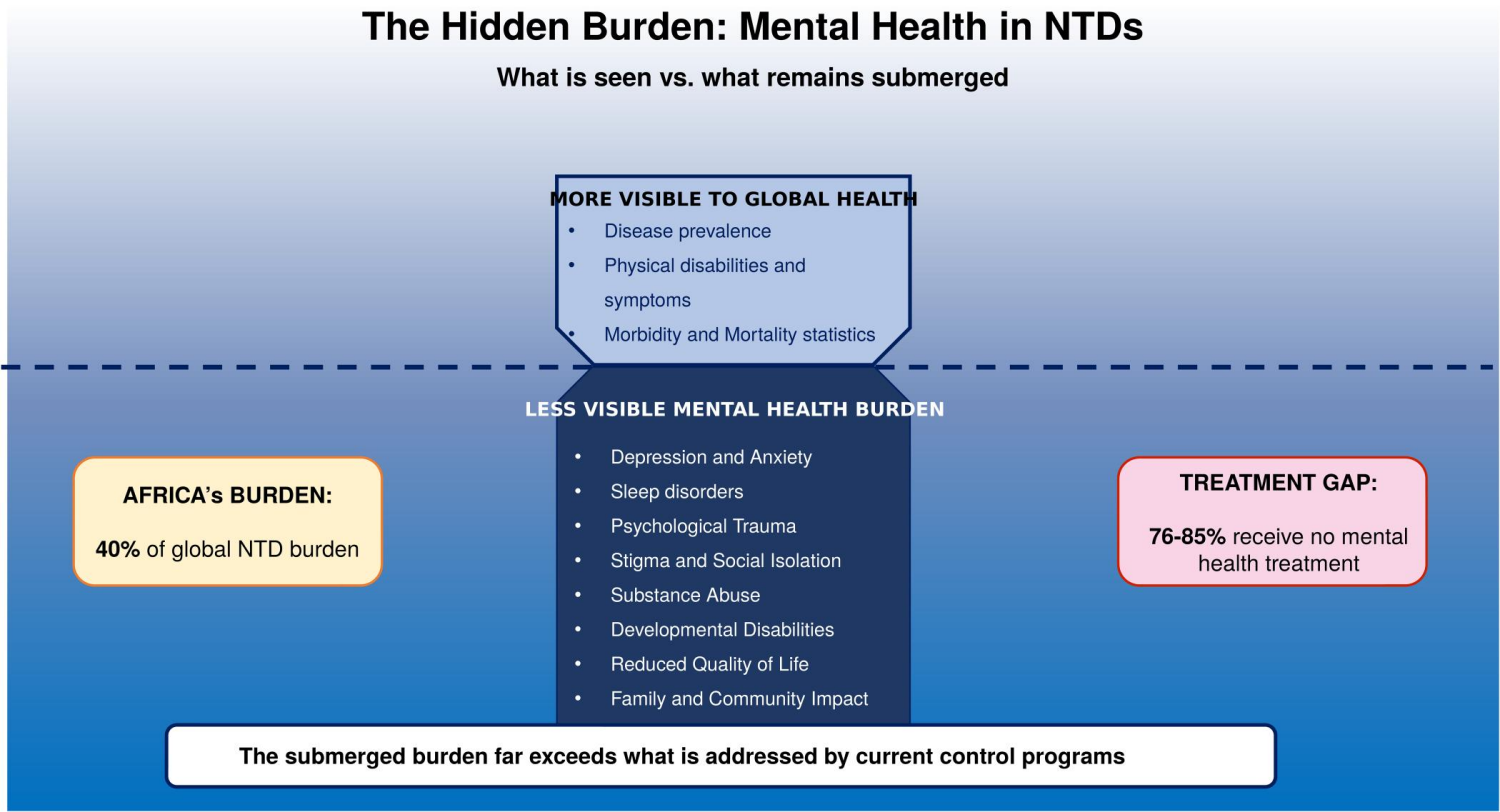
The “iceberg model” illustrates the disparity between the more visible and the less visible health burdens in NTDs. Above the waterline are the physical manifestations that receive attention in global health programming: physical disabilities, mortality statistics, and disease prevalence data. Below the waterline lies the far larger, hidden mental health burden affecting NTD patients, including depression and anxiety, post‐traumatic stress disorder (PTSD), psychological trauma, stigma‐related social isolation, substance abuse, developmental disabilities, reduced quality of life, and cascading impacts on families and communities. Africa bears a disproportionate 40% of the global NTD burden, with over 600 million people requiring treatment, predominantly in the poorest and most marginalized communities. Despite this enormous burden, an estimated 76%‐85% of people with mental health disorders in low‐ and middle‐income countries (LMICs) receive no treatment for their conditions. This “neglect within neglect”: the systematic exclusion of mental health considerations from NTD control programs, represents a critical failure of the “health for all” vision, undermines health equity, and threatens achievement of Sustainable Development Goal 3 (SDG3). The submerged portion of the iceberg, representing the unaddressed mental health burden, is not yet systematically captured in most disease burden assessments and intervention strategies.

Growing critiques of the current global health architecture have questioned whether current approaches and strategies genuinely serve the interests of those most affected by disease. The recurring question, “What is wrong with global health?”, voiced by different stakeholders, including scholars, practitioners, and affected communities, challenges the field to examine its priorities, power structures, and definitions of success [4, 5]. The question rarely comes with a single holistic answer. It rather amplifies an equally important counter‐question, “What is right with global health?” Much like the parable of the blind men and the elephant, each observer perceives only a part of the complex reality. This Viewpoint examines one such blind spot (the mental health burden of NTDs) through an African lens, given the enormous disproportionate burden of the disease and our grounding as African researchers, to illustrate how global health's narrow focus primarily on physical outcomes leads to the perpetuation of health inequities. We argue that the systematic neglect of mental health morbidity in NTD programs represents a critical failure of the “health for all” vision that undermines health equity and threatens the achievement of Sustainable Development Goal 3, with particularly severe consequences for Africa and other resource‐limited settings.

An “African lens,” as used here, refers to a standpoint that emphasizes the structural, historical, and sociocultural determinants of health inequity on the continent, rather than treating Africa merely as a site for implementing globally designed interventions. Critiques of global health have argued that the field's institutions, funding flows, and research agendas remain disproportionately shaped by high‐income actors, so that African disease burdens are addressed through externally defined priorities rather than locally grounded understandings of illness, disability, and wellbeing [5]. Within the NTD control agenda specifically, this has translated into a narrow biomedical focus on parasitological cure and physical morbidity, while the psychosocial and socioeconomic dimensions of living with a disfiguring or stigmatizing condition (dimensions that are often central to how affected communities themselves define the harm caused by these diseases) are either treated as secondary or absent from program design [6]. Applying an African lens, therefore, means not only asking which mental health services could be added to current NTD control programs, but why mental health was excluded from their design in the first place, and whose definitions of health and success have shaped that omission.

In addition, the African lens also requires attention to how the exerted burdens intersect rather than simply co‐occur. Women and girls disfigured by NTDs often face compounded disadvantage: physical disability limits economic opportunity, while visible disfigurement independently reduces marriageability and social standing in ways rarely experienced by men with the same condition [7]. Poverty intensifies this cycle, since the rural, low‐income communities most affected by NTDs also have very limited access to mental health services [8], so gender, disability, and poverty reinforce one another rather than acting as separate burdens.

Although this analysis is foregrounded through an African lens, given the continent's disproportionate NTD burden and our positionality, it is important to note that the proposed lens is not exclusive to Africa. Rather, several core features (including health priorities that are often externally defined, biomedical framings that bypass local understandings of illness and disability, and chronic lack of or underinvestment in the psychological and social dimensions of disease) are shared by other historically peripheralized regions, including Latin America and the Pacific, as well as parts of South Asia, and, where NTDs similarly intersect with limited mental health infrastructure [5, 9]. While, therefore, we present the African lens in one instance, we also present a broader decolonial critique of global health that can be adapted to other non‐Western settings faced with comparable structural neglect, with appropriate contextual grounding.

## 2 NTDs and Mental Health: Epidemiology, Burden, and Connection

NTDs represent a subset of infectious diseases caused by biologically disparate groups of pathogens, including vector‐borne protozoa, bacteria, fungi, nematode worms, soil‐transmitted helminths (STHs), non‐tuberculosis mycobacteria, and snakebite envenomation [10, 11]. These diseases affect the world's poorest, most vulnerable, and most marginalized communities, predominantly in 149 countries across Africa, Asia, and the Americas [12], causing illness, suffering, impairment, stigma, and approximately 200,000 deaths per year [13, 14]. The World Health Organization (WHO) currently recognizes 21 major NTDs (up from the 13 initially identified), which together affect approximately 1 billion individuals, including about 500 million children [14]. Critically, NTDs disproportionately affect women, girls, children, and the elderly population in terms of exposure, burden, and morbidity due to biological differences, gender roles, and family dynamics [7, 15, 16].

Beyond the associated direct morbidity and mortality, NTDs increase susceptibility to and perpetuate a wide range of communicable and non‐communicable diseases, including cervical cancer [17], bladder cancer [17], liver cirrhosis [18], and HIV/AIDS [19]. Due to their severe immunosuppressive effects, recent research suggests that NTDs can also worsen the prognosis for people affected by the three major global public health threats prioritized by the Global Fund: HIV/AIDS, tuberculosis (TB), and malaria [20, 21]. This evidence raises the question of whether the Global Fund should continue to focus on the so‐called ‘big three’ while neglecting NTDs [22]. Indeed, the UK Government's recent funding cut of over £150 M to programs supporting NTD research and implementation (in 2021) [23], and recent cuts by the US government, further exemplify this neglect and threaten to reverse hard‐won gains achieved over the decades [24]. When observed through an African lens, these funding withdrawals are not accidental: they highlight how the priorities for disease control on the continent continue to be highly influenced by external donor governments and other funders, rather than by the local health systems and communities bearing the disease burden, reinforcing the very dynamic of externally defined priorities previously described.

Despite substantial research on the burden exerted by NTDs, mental health research among those infected remains profoundly neglected [25]. Pooled prevalence of depression in lower‐limb lymphoedema‐related NTDs (leprosy, podoconiosis, lymphatic filariasis) is estimated at around 38% [26], with individual studies reporting anywhere from roughly 10% to over 70% depending on disease and setting [27]. Though emphasized a few years ago in a WHO report captioned ‘Mental health of people with neglected tropical diseases' [28], urgent action is needed by all stakeholders to move beyond policy development to implementation. According to the WHO, mental health is “a state of mental well‐being that enables people to cope with the stresses of life, realize their abilities, learn well and work well, and contribute to their community” [29]. Poor mental health is associated with relative social disadvantage in whole populations and vulnerable subgroups [30]. Unlike NTDs, which are more prevalent in low‐ and middle‐income countries (LMICs), mental health disorders are one of the leading causes of disability worldwide [31]. The Global Burden of Disease study (2023) estimated that 792 million people were living with mental health disorders in 2023 [32]. A 2014 systematic review estimated that approximately eight million deaths each year are attributable to mental disorders [33].

These disorders manifest differently but are generally characterized by a combination of abnormal thoughts, perceptions, emotions, behavior, and reduced social interaction [34]. Mental disorders span a spectrum from milder conditions like anxiety to more severe episodes of depression, psychological trauma, stigma, and schizophrenia [35]. Adverse conditions such as poverty, discrimination, and violence exert a powerful influence on mental health, especially in LMICs, where NTDs already impose a huge burden [36]. The WHO reports that health systems have not yet adequately responded to the burden of NTD‐associated mental disorders. Consequently, the gap between the need for treatment and its provision is very vast. Health systems in the LMICs face multiple challenges, including poor data quality, inadequate human resources, low management capacity, and technological difficulties, that hinder the adequate assessment of mental disorder burden [37]. More precarious is the fact that most LMICs, bearing the greatest brunt of the mental disorder burden, have neither adequate infrastructure to manage mental health disorders nor the appropriate policies for mental health promotion. Consequently, it is estimated that about 76%–85% of people with mental disorders in the LMICs receive no treatment for their condition [38]. In addition, for those who do receive any form of care at all, the quality remains questionable. Although current global mental health research and funding remain concentrated on high‐income settings, populations in LMICs bear the largest share of the combined NTD and mental health burden [39]. They are therefore central, not tangential, to the current drive towards achieving global health goals.

### 2.1 Global Health and NTDs: Science and Neglect

The important debate around a unified definition of global health has attracted extensive scholarly attention in recent decades [40]. Though several definitions exist, a common denominator among them has been their focus on “promoting health for all,” the vision adopted by the World Health Assembly in 1977 [41, 42]; and more recently expressed through the language of universal health coverage (UHC) [43]. Koplan et al., in 2009, defined global health as: “an area for study, research, and practice that places a priority on improving health and achieving health equity for all people worldwide” [44]. This definition, which encompasses the characteristics of equity (in health status and access), global conceptualization (as opposed to an international or supranational perspective, causes (of health issues), means (for health activities), and solutions (to address health issues), identified as fundamental components following an inductive analysis of selected key definitions [45], is the most widely accepted. A 2025 update to this definition further highlights equity, One Health, and planetary health considerations, reframing global health as a field that advances the equitable protection and improvement of population and planetary health [46]. It is, however, clear that the fight against NTDs is yet to be scrutinized through the different lenses included in this definition: persistent rarity in equity analyses of NTD burdens [3]; the field's means and causes continue to be defined primarily by external, high‐income actors and not by the locally affected communities [5]; and funding schemes like the Global Fund continue to not prioritize NTDs relative to the “big three” despite a comparable disease burden [22]

NTDs, despite receiving insufficient attention on the global health agenda, continue to exert a tremendous toll on global health, with a collective burden comparable to that of HIV/AIDS, tuberculosis, or malaria [12]. These diseases, which predominate in tropical regions, are called “neglected” not only because they generally afflict the world's poorest and most disadvantaged populations, but also because of previously reduced commercial and research incentives, weak political prioritization, and their exclusion from mainstream global health financing and metrics [12]. In the context of Africa, this exclusion is particularly conspicuous: ministries of health and research institutions from African countries remain underrepresented in setting research priorities and metrics for NTDs, even though the African population bears the greatest share of global NTD burden. This further mirrors the broader pattern of global health priorities heavily defined externally, as earlier introduced. In addition, NTDs are the leading causes of chronic disability and poverty in the LMICs; they are less well known and historically have not received as much visibility compared to other frequent non‐communicable diseases in high‐income countries. The devastating effect of these diseases on health and development, alongside evidence that their global burden surpasses other chronic diseases, reinforces the imperative for strengthening primary health care. Despite this, financial support for control and elimination efforts, as well as research and development (R&D), remains inadequate [47]. Also, because of the class of the population affected by these diseases, a significant fraction of these diseases (particularly male/female genital schistosomiasis, noma, mycetoma, neurocysticercosis, podoconioisis, snake envenomation, leprosy, etc.) have been largely ignored by drug manufacturers and public policymakers for decades. Consequently, the control of NTDs remains inadequate and extremely difficult today [48].

Though the importance of NTDs has been increasingly recognized more and more in the context of current global health goals (e.g., with the formal adoption of January 30th as World NTD Day during the 74th World Health Assembly in May 2021) [49], much remains yet to be done. Control programs at the local, national, and international levels have thus far failed to incorporate the necessary components for effective delivery. If the slogan, “*No health without mental health*,” [50] is true, which it undoubtedly is, the question that remains unanswered is: “Where in the global health agenda is the mental health morbidity of NTD sufferers properly addressed, given that this group of diseases causes significant disfigurement, debilitation, stigma, and disability?”

## 3 The New Neglect in NTDs: Mental Health

The WHO Constitution, which came into effect on April 7, 1948, defines health “as a state of complete physical, mental, and social well‐being and not merely the absence of disease or infirmity” [51]. NTDs impact all the aspects of well‐being cited in this WHO definition, at both the individual and community levels [7]. As summarized in Table 1, the physical manifestations and social repercussions of NTDs create a direct pathway to a range of mental health conditions, which are systematically overlooked by current control strategies.

**Table 1:** The vicious cycle of neglect: interconnections between NTD morbidity, mental health consequences, and systemic gaps.

NTD‐related morbidity/stressor	Examples of associated mental health consequences	Current gaps in NTD programmatic response
**Disfigurement and disability** (e.g., Elephantiasis, limb loss from leprosy, blindness)	Depression, anxiety, social phobia, post‐traumatic stress, low self‐esteem	Focus on preventive chemotherapy; no psychosocial support is rarely integrated into Mass Drug Administration (MDA) programs.
**Stigma and social exclusion** (e.g., Rejection, loss of employment, educational barriers)	Isolation, loneliness, major depressive disorders, substance abuse	Lack of community sensitization and stigma‐reduction programs linked to NTD control.
**Chronic pain and debilitation** (e.g., from neuropathic pain, chronic inflammation)	Anxiety, depression, sleep disorders, reduced quality of life	Health systems lack capacity for chronic pain management and mental health screening.
**Poverty and financial hardship** (Due to care costs and lost productivity)	Hopelessness, stress, anxiety disorders	NTD programs are vertically focused and not integrated with social protection or livelihood support.
**Neurological sequelae** (e.g., from neurocysticercosis, snake envenomation)	Cognitive impairment, psychosis, organic mood disorders	Lack of neurological and psychiatric expertise within NTD control programs.

Many NTDs have long‐term debilitating consequences, including visual, physical, and social impairments [52]. Physical conditions such as elephantiasis (in lymphatic filariasis and podoconiosis), loss of limbs in leprosy, and visual impairments/blindness in onchocerciasis and trachoma represent just a fraction of the plethora of disabilities suffered by affected persons. These disabilities tremendously affect the lives of sufferers in terms of personal and family productivity while also causing stigma and discrimination, leading to, amongst others, social exclusion, rejection, reduced access to health and social services, lack of educational opportunities, exclusion from income‐generation and employment opportunities, restrictions on civil and political rights, and reduced quality of life [7]. In addition, several NTDs, including onchocerciasis, trichuriasis, and schistosomiasis, are associated with delayed physical and mental development and developmental disabilities that have been largely overlooked [53]. The combined effects of this burden contribute to and aggravate the mental health status of affected persons [52], resulting ina self‐perpetuating cycle (Figure 2) that includes disorders such as substance abuse and also increasing susceptibility to nutritional and metabolic diseases, cardiovascular diseases, viral diseases, respiratory tract diseases, musculoskeletal diseases, sexual dysfunction, pregnancy complications, stomatognathic diseases, and possibly obesity‐related cancer, further driving mortality amongst those affected [54]. Yet, the mental burden of NTD sufferers has continuously been ignored. This raises the question of how well NTD control programs can perform if the mental health burden exerted on sufferers is not given due consideration.

**Figure 2:**
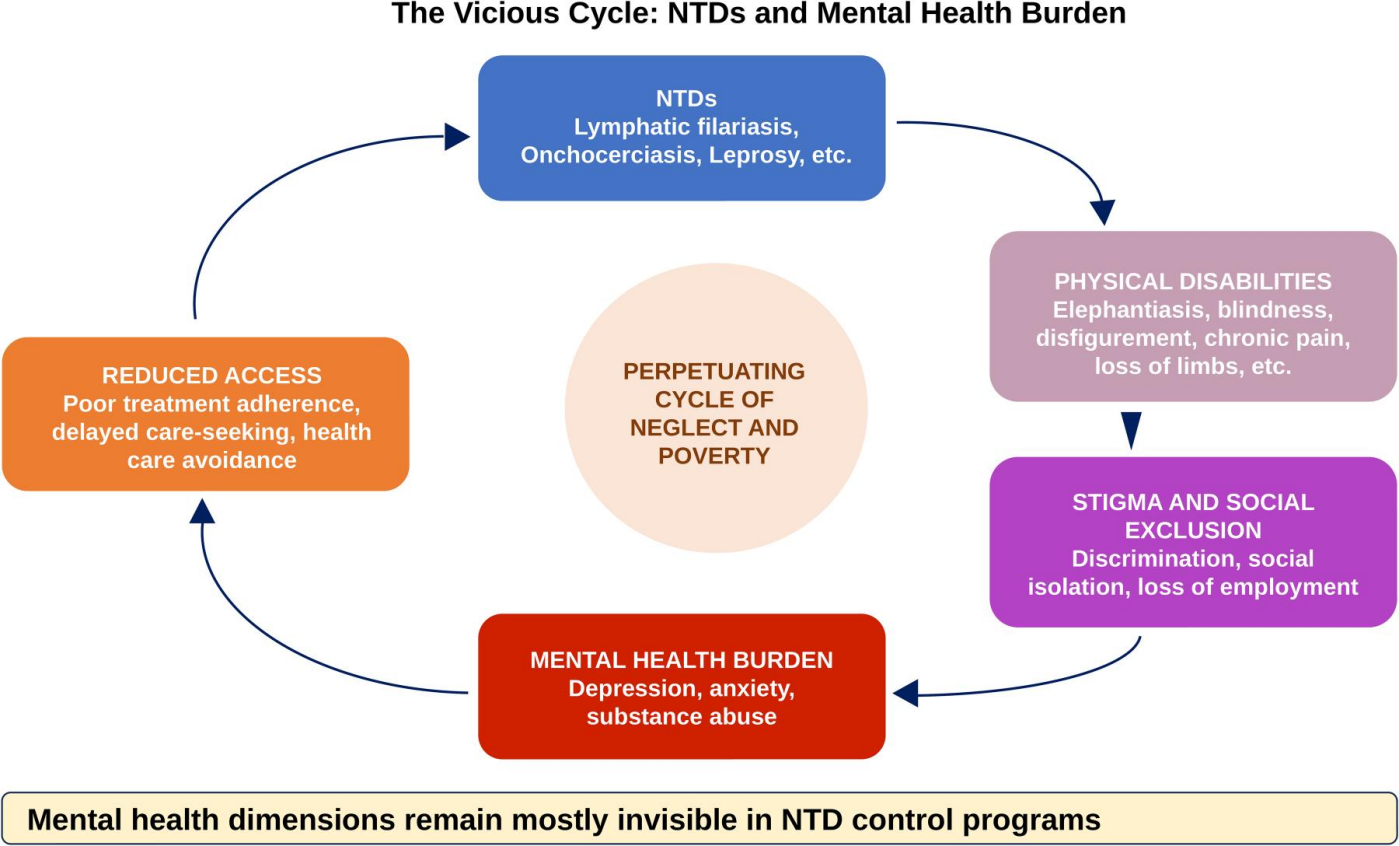
The vicious cycle perpetuating NTDs and mental health burden. The diagram illustrates the self‐reinforcing cycle linking NTD infections with mental health disorders: a cycle that remains unaddressed in current control strategies. NTDs (including lymphatic filariasis, onchocerciasis, leprosy, schistosomiasis, and others) lead to severe physical disabilities, such as elephantiasis, blindness, disfigurement, chronic pain, and limb loss. These visible disabilities trigger profound stigma, social exclusion, discrimination, isolation, and loss of employment opportunities. The combined physical and social burdens contribute to and exacerbate mental health disorders, including depression, anxiety, PTSD, and substance abuse. Mental health conditions, in turn, reduce treatment adherence, delay care‐seeking behavior, and promote healthcare avoidance, ultimately perpetuating NTD transmission and worsening health outcomes at both individual and community levels. This vicious cycle is particularly devastating in African and other LMIC settings where mental health infrastructure is severely limited or absent. Despite the central role of mental health in this cycle, the mental health dimension has remained largely invisible in NTD control programs, which currently focus principally on five strategies: preventive chemotherapy, intensified disease management, vector control, veterinary public health measures, and improved water and sanitation. Breaking this cycle requires integrated psychosocial support alongside existing biomedical interventions and others. The bottom banner emphasizes that without addressing mental health, global targets for NTD elimination and management will likely remain unattainable, perpetuating inequities in the world's most vulnerable populations.

## 4 Sustainable Development Goals (SDGs), NTD Roadmap, and Recommendations

Mental health conditions remain one of the most neglected areas of health globally and are predicted to be the largest contributor to the global health burden by 2030 [55]. This neglect is most acute and pronounced in LMICs lacking adequate infrastructure to cater to sufferers' needs, especially for persons affected by one or more NTDs. More than 85% of the world's population lives in the 153 LMICs, which harbor almost all those afflicted by NTDs. Additionally, more than 80% of persons with mental disorders reside in LMICs [56], revealing a significant overlap with NTDs. This overlap underscores the need for a more comprehensive and holistic approach to controlling NTDs, most of which are associated with mental disorders ‐ especially depression. Several NTDs also have neurological sequelae, indicating that NTDs can have a direct and causative effect on mental disorders [52].

However, more neglected (in terms of infrastructure, funding, research, policy development and uptake, and public health attention) than NTDs is their associated mental health impact. For instance, mental health disorders (e.g., anxiety, depression, and others) are still not being adequately considered when assessing the burden related to NTDs. With the current programmatic shift from control to elimination in the global health goal of several NTDs and in line with SDG3, which focuses on, amongst others, the “big three” and NTDs, the WHO published the NTD Roadmap 2021–2030, defining global targets and milestones to achieve various NTD‐specific agendas. Interestingly, this document acknowledges the intricate connection between NTDs and SDGs in its title, “**ENDING the NEGLECT to ATTAIN the SUSTAINABLE DEVELOPMENT GOALS.**” The shift in programmatic goals calls for a commensurate shift in the paradigm regarding the control strategies put in place.

NTDs are typically addressed through five strategies: preventive chemotherapy, intensified disease management, vector control, veterinary public health measures for zoonotic neglected diseases, and improved water and sanitation [57]. Currently, none formally incorporates the mental health dimension of NTDs, although isolated national programs report ad hoc psychosocial components. Due to the high psychosocial impact of NTDs on sufferers, global targets for elimination and management will likely remain unattainable without integrated psychosocial support to complement present strategies. Though challenges such as geographical inaccessibility, insecurity, and weak health systems continue to prevail in the poorest NTD‐endemic countries, substantial progress can be made in promoting mental health programs in NTD‐endemic communities through multi‐dimensional, cross‐cutting approaches involving diverse stakeholders.

At the core of successful global NTD control is community involvement under the leadership of health ministries in endemic countries. This is evident from how community‐directed treatments with ivermectin (CDTI) have strengthened health systems for onchocerciasis control, and how universal test‐and‐treat systems have reshaped timely HIV/AIDS management in district settings [47]. Similar community‐based approaches can mitigate mental health burdens associated with NTDs. These models represent what an African lens asks for, practically: interventions that are designed around existing community structures and locally grounded and defined needs, rather than imported, top‐down programming. They provide a template for how the integration of mental health into NTD control could similarly be community‐guided and ‐led rather than externally imposed.

At the national control program level, incorporating mental health components into NTD control agendas is essential. Equally important are operational support structures to meet the ever‐increasing mental health and social needs of endemic communities, including:

- Community health worker training in validated brief screening tools (e.g., the WHO mhGAP Intervention Guide, PHQ‐9 for depression and WHODAS 2.0 tool for disability) to identify and refer NTD patients with mental health needs, alongside basic psychosocial support, integrated into existing physical therapy and counseling services (similar to HIV/AIDS management)
- Education and sensitization sessions for community leaders and members on stigma and discrimination reduction
- Training on employment opportunities for affected persons through local productive skills acquisition
- Social participation programs for people with disabilities to foster community integration
- Provision of psychosocial support to NTD sufferers, especially those with physically debilitating symptoms like nodules, hanging groin, and others.

These interventions, which can be integrated into mass drug administration campaigns, could tremendously contribute to improving the sense of worth of NTD sufferers.

Historically, many donor agencies and research organizations have focused principally on developing tools such as diagnostics, vaccines, novel chemotherapeutics, and implementation research. To enable greater researcher engagement with NTD‐associated mental disorders, increased funding must be made available, especially in the sub‐Saharan African region and other LMICs where these disorders are most prevalent. In addition, there is a dire need for NTD researchers to collaborate with mental health researchers and acquire training and skills in using mental health research tools. Harnessing these efforts through collaborative synergies with different actors, resources, and institutions would substantially reduce the NTD‐associated mental health burden and contribute to meeting the goals set in the NTD 2021‐2030 Roadmap and SDG3. This would represent meaningful progress in addressing what is wrong with global health. Beyond the geographical limits of the African continent, the approach outlined here (highlighting locally grounded definitions of harm, giving attention to the intersecting structural disadvantage, and questioning whose and which priorities should shape disease control agendas, especially in the LMICs) offers a valuable and transferable template for more research work into neglected comorbidities in other historically peripheralized regions. Our encouragement to researchers working in and beyond African contexts is to apply similarly critical, structurally attentive lenses to other blind global health spots where, though biomedical progress is being claimed and reported, persistent and unmeasured burdens continue to be exerted on affected communities.

## Author Contributions

**Robert Adamu Shey:** writing – original draft, writing – review and editing, conceptualization, formal analysis, visualization. **Gordon Takop Nchanji:** conceptualization, writing – review and editing, writing – original draft, formal analysis, visualization. **Yanick Aqua Stong Tangan:** writing – review and editing, visualization. **Melvis Berinyuy Dulandzev:** writing – review and editing, writing – original draft. **Methodius Shinyuy Lahngong:** writing – review and editing, writing – original draft. **Francis Nongley Nkemngo:** formal analysis, writing – review and editing, visualization, writing – original draft. **Cabirou Mounchili Shintouo:** writing – review and editing, visualization, writing – original draft. **Ntang Emmaculate Yaah:** writing – review and editing, visualization. **Bernis Neneyoh Yengo:** writing – review and editing, visualization. **Stephen Mbigha Ghogomu:** writing – review and editing, supervision, methodology, conceptualization, project administration, resources, visualization.

## Funding

The authors have nothing to report.

## Ethics Statement

The authors have nothing to report.

## Conflicts of Interest

The authors declare no conflicts of interest.

## Transparency Statement

All authors have read and approved the final version of the article. Robert Adamu Shey, the corresponding author, had full access to all of the data (literature and sources cited) in this study and takes complete responsibility for the integrity of the data and the accuracy of the data analysis. Robert Adamu Shey affirms that this article is an honest, accurate, and transparent account of the argument being reported; that no important aspects have been omitted; and that there were no discrepancies from the article as planned.

## Data Availability

Data sharing is not applicable to this article, as no datasets were generated or analysed during the current study.
